# Editorial: Shaping the future of predictive toxicology: addressing challenges and new approach methodologies

**DOI:** 10.3389/fphar.2026.1978373

**Published:** 2026-09-29

**Authors:** Gladys Ouedraogo, Elisabet Berggren, Thomas Hartung

**Affiliations:** 1 Research and Innovation, L’Oréal, Aulnay-sous-Bois, France; 2 European Commission, Joint Research Centre (JRC), Ispra, Italy; 3 Center for Alternatives to Animal Testing (CAAT), Bloomberg School of Public Health, Johns Hopkins University, Baltimore, MD, United States; 4 CAAT-Europe, University of Konstanz, Konstanz, Germany

**Keywords:** 3Rs, adverse outcome pathways (AOP), machine learning, new approach methodologies (NAMs), predictive toxicology, quantitative systems toxicology, regulatory science

## Introduction

Toxicology is being remade. Over the past three decades, New Approach Methodologies (NAMs) (the collective term for *in vitro*, *in silico*, *in chemico*, and other non-animal tools together with the data-integration frameworks that give them meaning) have moved from the periphery of safety science toward its center. The 2013 European ban on animal testing for cosmetics turned a scientific aspiration into a regulatory necessity, and the momentum has since spread across pharmaceuticals, food and feed, agrochemicals, and industrial chemicals. Regulatory agencies on both sides of the Atlantic have signaled that the phase-out of routine animal testing is no longer a question of whether, but of how and how fast. The convergence of artificial intelligence (AI), computational modeling, high-content *in vitro* systems, and organ-on-chip engineering has widened the space of what is measurable and predictable, while simultaneously raising the bar for what must be qualified, validated, standardized, and trusted before a method can inform a regulatory decision.

This Research Topic was conceived to capture that transition in motion. It brings together 21 contributions from more than 130 authors across academia, industry, and regulatory bodies, published in *Frontiers in Pharmacology*, *Frontiers in Toxicology*, and *Frontiers in Immunology*. The Research Topic is deliberately heterogeneous and covers several sectors. It spans from purely computational hazard predictors, mechanistic virtual-tissue models, human-relevant *in vitro* platforms, adverse outcome pathway (AOP) frameworks, fish embryo models, to AI-enabled pharmacovigilance, and classical mechanistic and clinical toxicology. Read together, these papers do not describe a finished paradigm so much as a working one: a field assembling, method by method, the evidence base and the trust that a predictive, human-relevant toxicology will require. In this editorial we group the contributions thematically and reflect on what they collectively signal about the road ahead.

## Computational prediction: from chemical structure to hazard

The most mature NAMs are computational, and several contributions show both their promise and their remaining limits. Khan et al. built an ensemble model combining machine learning and deep learning to predict hepatotoxicity across a dataset of more than 2,500 compounds; their best voting ensemble, built on Morgan fingerprints, reached an accuracy above 80% and outperformed published benchmarks, while notably extending the training set beyond drugs to industrial chemicals. Tong et al. addressed a subtler but critical problem in structure-based prediction: how much confidence to place in the targets that similarity-centric “target-fishing” models return. By systematically evaluating fingerprints, scoring schemes, and crucially fingerprint-specific similarity thresholds, they showed that the similarity score itself can serve as a quantitative measure of reliability, a prerequisite for using such tools in off-target and adverse-effect prediction. Huang et al. illustrated where computational toxicology is heading: an integrated pipeline combining network toxicology, machine learning with SHAP-based interpretability, and molecular docking to identify ERBB2-associated inflammatory signaling as a candidate mechanism by which the plasticizer metabolite mono-(2-ethylhexyl) phthalate promotes hepatic lipid accumulation, with the computational predictions then confirmed in human liver cell lines rather than in animals.

## Quantitative systems toxicology and virtual tissues

A second cluster moves from statistical prediction toward mechanistic, physiologically grounded simulation. Gupta et al. coupled a physiologically based pharmacokinetic (PBPK) model of valproic acid with a large-scale hepatocyte metabolism model to reproduce drug-induced steatosis, demonstrating that a credible quantitative systems toxicology (QST) prediction required not only the metabolic inhibition step but also the regulatory feedback (PPARα signaling) that governs it; this is a reminder that mechanistic fidelity, not model size alone, drives predictivity. Ghosh et al. built a multiscale, zonation-resolved virtual model of the human liver lobule, coupled it to PBPK and acetaminophen-injury kinetics, and used four clinical overdose cases to dissect how the spatial zonation of metabolic parameters shapes dose-dependent hepatocellular necrosis, i.e., an early example of the “virtual patient” approach that regulators have begun to encourage for drug-induced liver injury. Complementing these mechanistic models, Duchateau-Nguyen et al. tackled a different lever of the 3Rs: whether virtual control groups, assembled from a large historical repository of rat control animals, can substitute for concurrent controls. Across 40 studies, virtual and concurrent controls agreed fully in nearly half of comparisons and disagreed fully in only a small minority, with a hybrid partial-replacement strategy performing best; this is a concrete route to reducing animal use in preclinical toxicology without materially altering study conclusions.

## Human-relevant *in vitro* systems and the discipline of standardization

NAMs are only as good as their reproducibility, and three contributions foreground the unglamorous but decisive work of characterizing and standardizing *in vitro* systems. Jochum et al. showed that seemingly minor cultivation parameters (seeding density and time in culture above all) measurably shift cytochrome P450 gene transcription in HepaRG cells, with clear implications for the comparability of *in vitro* toxicological assessments across laboratories. Singh et al. developed mouse lung organoids as a three-dimensional platform for profibrotic toxicity, benchmarked against an *in vivo* cadmium fibrosis model and applied to drugs such as amiodarone and nitrofurantoin, extending predictive toxicology into pulmonary fibrosis where few validated NAMs exist. Spruck et al. contributed a resource of a different kind: HIDmap, a FAIR, interactive, physiology-based map of human immune system development spanning 24 organs and hundreds of cell types, explicitly designed to support animal-free developmental immunotoxicity research and embedded within an AOP framework. Together these papers make the point that human relevance is achieved not by any single clever assay but by the sustained curation, quality control, and interoperability that let assays be trusted and combined.

## Mechanistic frameworks: adverse outcome pathways (AOP) and regulatory translation

If NAMs are the components, AOPs and defined approaches are the architecture that turns them into decisions. Two contributions sit at this regulatory-science core. Sachana et al. reviewed roughly a decade of OECD-led work to develop the developmental neurotoxicity *in vitro* battery, tracing its journey “from concept to regulation” (the assembly of assays covering neural proliferation, migration, differentiation, and network formation, their integration into integrated approaches to testing and assessment (IATA)), and the honest catalogue of what remains: formal battery validation, metabolic competence, barrier tissues, and quantitative *in vitro*-to-*in vivo* extrapolation. Clerbaux et al. applied the same logic to an urgent, human-specific hazard: cytokine release syndrome from immunotherapies such as CAR-T cells and T-cell engagers, which animal models and conventional assays predict poorly. They proposed an immune-related AOP that organizes the syndrome around harmonized inflammatory key events and maps them onto human-relevant NAMs, from cytokine release assays to vascular organ-on-chip systems, while calling for the quantitative key-event relationships and next-generation risk-assessment integration that would make such a framework regulatory-ready. HIDmap’s AOP anchoring reinforces the same theme: mechanistic scaffolds are what let disparate human-based methods speak to one another and to regulators.

## Fish embryo models and image-based AI

Non-mammalian and AI-augmented models occupy an important middle ground between *in vitro* screening and mammalian testing. Two zebrafish studies exemplify it. Małkowska et al. used the zebrafish embryo to quantify the developmental toxicity of paracetamol and ethanol in combination, showing frank synergy (combination indices below one across effect levels) and underscoring that mixture effects, a persistent blind spot of single-compound testing, are tractable in alternative models. Majdan et al. went a step further by integrating three NAMs at once: *in silico* QSAR prediction, zebrafish embryotoxicity testing, and an attention-based convolutional neural network that classified embryo phenotypes automatically, applied to the food dye quinoline yellow. Their work is a compact illustration of where the field is converging: computational prediction, an alternative model, and AI-based readout woven into a single assessment.

## Data-driven pharmacovigilance and biomarker discovery

AI is reshaping not only prospective hazard prediction but also how we learn from human data after exposure. Wu et al. built interpretable machine-learning models to predict tigecycline-related hepatotoxicity and coagulation dysfunction from routine clinical features, achieving useful discrimination on external validation and delivering the results as a usable clinical nomogram with SHAP-based explanations. Tian et al. combined untargeted metabolomics with machine learning and SHAP analysis to nominate predictive biomarkers of immune-checkpoint-inhibitor rash, while candidly cautioning that near-perfect classifier performance on a small cohort may reflect overfitting, i.e., an honesty about generalizability that the field needs more of. Yang et al. mined the FDA Adverse Event Reporting System to characterize medication-induced lactic acidosis, quantifying disproportionality signals across more than a thousand drugs and flagging that many implicated medications carried no corresponding label warning. These studies show real-world and omics data becoming, in effect, a continuous validation stream against which predictive methods can be calibrated.

## Mechanistic depth from omics-informed and clinical studies

Finally, the Research Topic includes classical mechanistic and clinical toxicology, and it belongs here for a reason: predictive methods must ultimately reproduce the biology that such studies define. Mehari et al. paired histopathology with transcriptomic profiling to identify gossypol-responsive genes tied to fertility, nominating a hub gene through network analysis. Chen et al. used proteomics and metabolomics to map how an ethanol extract of *Emilia sonchifolia* injures the liver through cholestasis and oxidative stress, resolving the affected metabolic pathways. Yang et al. profiled the acute and subacute safety of baicalin under OECD guideline designs, establishing a no-observed-adverse-effect level. On the clinical side, Liu et al. compared chemotherapy-induced peripheral neuropathy between docetaxel and nab-paclitaxel in a prospective cohort, distinguishing their sensory-versus-motor phenotypes, and Liang et al. documented, via MRI, the toxic encephalopathy of a metoclopramide and multiple drug overdose. These contributions are not NAMs, but the molecular pathways, dose-response anchors, and human phenotypes they provide are precisely the ground truth that *in vitro* and *in silico* methods aspire to predict and against which their credibility will be judged.

## Challenges and outlook

Viewed as a whole, this Research Topic maps both the reach and the unfinished business of predictive toxicology. Several challenges recur across the contributions. Validation and standardization remain the rate-limiting step: assays as different as HepaRG monolayers and multiscale liver models are only decision-useful when their reproducibility and domain of applicability are defined, as Jochum et al. and the OECD battery review (Sachana et al.) both stress. Mechanistic completeness matters as much as computational power; Gupta et al. show a prediction failing until a regulatory feedback loop is added, and the DNT battery still lacks metabolic competence and barrier tissues. AI brings its own discipline: interpretability (SHAP recurs throughout), guarding against overfitting on small cohorts as Tian et al. warn, and the FAIR, well-curated data that HIDmap (Spruck et al.) exemplifies. And human relevance (for immunotoxicity, mixtures, and idiosyncratic drug reactions alike) repeatedly exposes the limits of animal models and argues for human-based systems anchored in AOPs.

The trajectory, however, is unmistakable. Prediction is shifting from correlation toward mechanism; from single assays toward integrated, weight-of-evidence approaches; from animal surrogates toward human-relevant systems; and from static hazard lists toward dynamic, quantitative, patient-contextualized simulation. Realizing that future will require continued investment in reference data, transparent and governed AI, and above all the collaborative validation that moves a promising method into regulatory practice. The breadth of this Research Topic, and of the community it represents, gives grounds for confidence. We thank all the authors, reviewers, and the Frontiers editorial team for their contributions, and we invite readers to treat these papers not as endpoints but as waypoints on a road the whole field is building together.

